# Asbestos and Autoimmunity: More Bad News from Libby?

**Published:** 2005-01

**Authors:** Rebecca Renner

Autoimmune diseases such as rheumatoid arthritis, multiple sclerosis, and systemic lupus erythematosus seem to be the product of a complex and poorly understood interaction between environmental exposures and genetic predisposition. Autoantibodies may be markers of subclinical disease, so epidemiological studies that look for autoantibodies in populations exposed to likely environmental triggers offer one possible way to better understand this gene–environment interaction. To study whether asbestos could be such an environmental trigger, Jean Pfau and colleagues at the University of Montana in Missoula went to the nearby town of Libby, where they found evidence that asbestos exposure may indeed induce autoimmunity **[*EHP* 113:25–30]**.

Asbestos exposure in Libby stems from the mining of vermiculite, which is used for insulation and fireproofing. The vermiculite, mined extensively from the 1920s to 1990, was laced with toxic amphibole asbestos, and the mining operations released asbestos into the air and contaminated the mine, processing sites, and many of the buildings and properties in town. Homes also became polluted through the use of vermiculite for insulation and garden fill, according to U.S. Environmental Protection Agency investigations. Virtually the entire town was designated a Super-fund National Priorities List site in October 2002.

The decades of occupational and environmental exposure to amphibole asbestos in Libby have been linked to a high incidence of asbestos-related diseases including fibrosis, pleural plaques, and cancer. Anecdotal evidence suggests there may also be a link in Libby between asbestos exposure and autoimmunity. In a 2000–2001 screening of 7,307 Libby area residents by the Agency for Toxic Substances and Disease Registry, 6.7% reported having been diagnosed with an autoimmune disease. Pfau and colleagues note that figure typically should be less than 1%.

In the current study, the researchers sampled the blood of 50 middle-aged men and women from Libby and 50 matched controls from Missoula, where there is no known asbestos exposure. The samples were analyzed for antinuclear antibodies (ANAs) using a commercially available indirect immunofluorescence test. ANAs are a class of autoantibody often found in the blood of people whose immune systems may be predisposed to cause inflammation against their own body tissues. The researchers also looked for correlations between length of asbestos exposure, presence of asbestos-related disease, and ANA levels among the Libby subjects.

They found that ANAs occurred 28.6% more frequently in the Libby samples than in those from Missoula. This finding is consistent with the results of a limited number of other studies of populations exposed to asbestos. In addition, individuals who had been exposed to asbestos for more than five years tended to have higher concentrations of ANAs than those with less exposure. Of the people from Libby, 12 had no lung abnormalities, but the rest had asbestos-related lung problems; those with more severe lung problems also had higher concentrations of autoantibodies.

Based on the correlation between asbestos-related disease and ANA levels, the results suggest that asbestos is an agent of systemic autoimmunity and that autoimmune responses may play a role in the progression of asbestos-related diseases, according to the authors. Pfau and colleagues intend to continue their studies of actual autoimmune diseases among the Libby population.

## Figures and Tables

**Figure:**
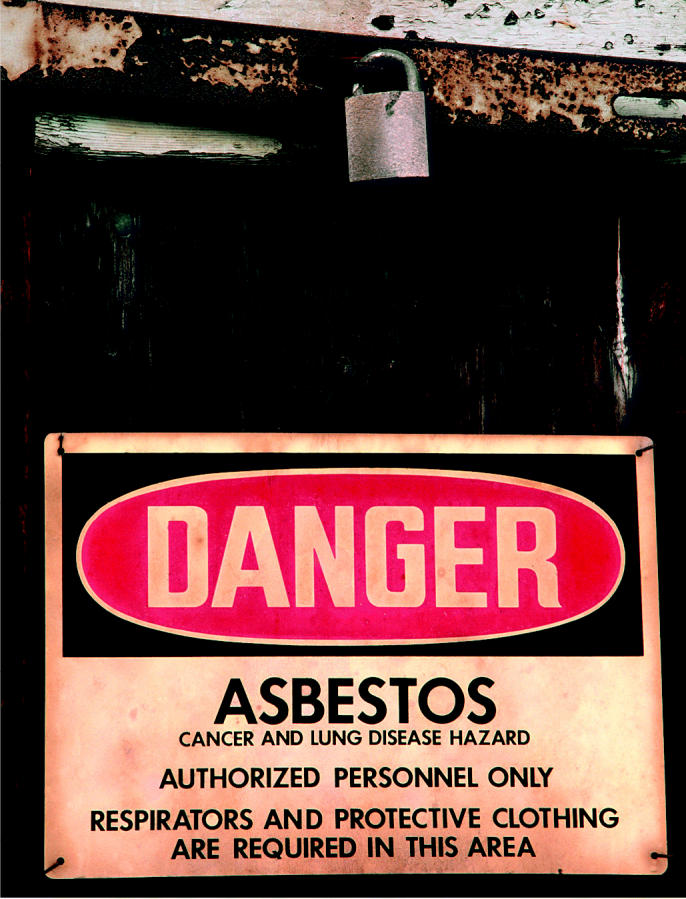
**Libby, Libby, Libby.** The saga continues for the residents of Libby, Montana, as new research suggests that amphibole asbestos exposure may induce autoimmunity.

